# Medical Fees in America

**Published:** 1839-04

**Authors:** 


MEDICAL FEES IN AMERICA.

The following scale of charges, for professional services, was adopted by the Washington County (N. Y.) Medical Society in June, 1837.

| | Doll. | Cts. |
|---|---|---|
| Advice at office . . | | 50 |
| Venesec., Ext. Dent., Cath. Emet., each . . | | 25 |
| Ordinary visit, under one mile | | 50 |
| For each additional mile, extra . . . | | 25 |
| Nocturnal visit extra | | 50 |
| Detention, per hour ,, | | 25 |
| Consultation ,, | 2 to 5 | 0 |
| Obstetrics, ordinary, not over six hours . . | 4 | 0 |
| Difficult—extraordinary cases—discretionary | | |
| Catheter, single introduction | 2 | 0 |
| ,, each succeeding . | 1 | 0 |

| | Doll. |
|---|---|
| Fracture, thigh and leg . | 5 to 10 |
| ,, all others . . | 2 to 5 |
| Compound do., extra—discretionary | |
| Dislocation, hip . . | 10 to 25 |
| ,, all others . | 3 to 10 |
| Compound do., extra—discretionary | |
| Amputation of large extremities . | 25 |
| Vaccination, single patient . | 1 |
| Paracentesis . . | 5 to 10 |
| Hernia, reduction by taxis . | 2 |
| ,, ,, by operation | 20 |
| Trepanning . . | 20 |
| Lithotomy . . . | 50 |

*Boston Med. and Surg. Journal. Feb.* 21, 1838.

